# From polycrisis to metacrisis: harnessing windows of opportunity for renewed political leadership in global health diplomacy

**DOI:** 10.1136/bmjgh-2024-015340

**Published:** 2024-04-18

**Authors:** Brian Li Han Wong, Anders Nordström, Peter Piot, Helen Clark

**Affiliations:** 1 International Politics, Leadership & Diplomacy for Health Project, Stockholm School of Economics and Karolinska Institute, Stockholm, Sweden; 2 Center for Resilient Health, Stockholm School of Economics, Stockholm, Sweden; 3 Department of Global Public Health, Karolinska Institute, Stockholm, Sweden; 4 Department of International Health, Maastricht University Care and Public Health Research Institute, Maastricht, Limburg, Netherlands; 5 European Union, Brussels, Belgium; 6 London School of Hygiene and Tropical Medicine, London, UK; 7 Helen Clark Foundation, Auckland, New Zealand

**Keywords:** Global Health, Health policy, Public Health, Decision Making, Accountability

**Summary box**

- Effective global health diplomacy necessitates multidisciplinary leaders skilled in navigating today’s complex health political landscape through innovative strategies and collaboration.
- The future of global and regional health advancement hinges on the investment in a new generation of leaders based on dynamic mentoring and learning methodologies.
- Historical achievements, underscored by strong political leadership, serve as a blueprint for overcoming major public health challenges through concerted action and political commitment.
- The COVID-19 pandemic has underlined the global vulnerability to infectious diseases, highlighting the imperative for international collaboration in health preparedness and response.
- To address global health challenges, leaders must engage across sectors, combining public health evidence and private sector perspectives with insights into various disciplines to effectively communicate and negotiate within the political sphere.

## Introduction

In an era marked by multifaceted and inter-related crises, global health stands at a crossroads of unprecedented challenges and emerging opportunities. The lingering effects of the COVID-19 pandemic, global health threats (such as the silent pandemic of antimicrobial resistance and the escalating threat of climate change), widespread geopolitical turmoil, rising populism, diminished global political leadership, persistent poverty and extreme deprivation, widening economic inequality and instability and mass displacement converge in a ‘polycrisis’.^1^ This confluence not only strains international cooperation but also diverts attention away from critical health priorities, undermining public confidence and eroding trust in public health systems and global health governance.^2^ Against this backdrop, the strategic importance of investment in health to secure economic and political stability as well as in safeguarding human health and well-being, cannot be overstated.

Following a recent conference on ‘International Politics, Leadership and Diplomacy for Health’ (held in Stockholm, Sweden, from 20 to 22 November 2023), a network of partners seeking to advance global health diplomacy was established. Coauthored by members of this network, this commentary emphasises crucial insights gained from the event and underscore the importance of identifying windows of opportunity as well as the urgency of reshaping global health and the leadership which underpins it. Moreover, it calls for a reinvigorated approach to health diplomacy infused with strong political leadership and innovative strategies to navigate the intricate, ever-evolving landscape of global health.

## Historical context and lessons learned

Historically, global health initiatives, driven by robust, government-led political leadership, have demonstrated significant success. Governmental convening and support were pivotal in the eradication of smallpox, the Framework Convention on Tobacco Control, the Pandemic Influenza Preparedness Framework and the Political Declaration of the High-Level Meeting of the General Assembly on the Prevention and Control of Non-communicable Diseases, illustrating how concerted global action can significantly reduce major public health threats.^3^ The global response to the HIV/AIDS epidemic, catalysed by the establishment of global health entities such as UNAIDS; the Global Fund to Fight HIV/AIDS, Tuberculosis, and Malaria; PEPFAR; and Unitaid, transformed a once dire prognosis into significantly improved conditions for millions. Similarly, Gavi, the Vaccine Alliance, has drastically improved vaccine accessibility in lower income countries, directly impacting child mortality rates worldwide. These lessons are further underscored by the COVID-19 pandemic, which highlighted the global threat of infectious diseases and the critical need for collaboration in preparedness and response, reinforcing that no nation is immune to pandemics. Ultimately, the reality of these examples accentuates the importance of effective political leadership in elevating health issues on the international agenda, resulting in concrete enhancements to public health.

## Current challenges

Today, the context in which these successes were achieved has changed. The contemporary political environment—characterised by a mix of emerging and enduring powers, shifting geopolitical landscape and extreme polarisation of publics and politics both within and between nations—presents novel challenges for global health diplomacy.^4^ The advent of digital transformation, while offering innovative solutions, also brings new complexities in terms of data privacy, equitable access, disinformation, mental health and digital health literacy.^5^ The COVID-19 pandemic revealed systemic weaknesses in global health governance and inequities in resource distribution, highlighting the inadequacy and fragmentation of existing accountability and governance mechanisms in the face of a global health crisis.^6^

Reports and calls highlighting the need for a robust global surveillance system, equitable access to health technologies, better preparedness and response financing and high-level political engagement have emerged.^7^ Yet, the gap between recommendations and their implementation reflects a dissonance between acknowledged needs and political action. This disconnect underscores the necessity not only to understand but also to engage with the political realities of health, as relying on public health evidence and arguments alone is insufficient.^7^ Health leaders not only need to understand politicians, the private sector, activists and academic and multilateral institutions but also to be able to communicate and engage constructively with them.

## Evolving leadership in global health diplomacy

Effective global health diplomacy today demands a new generation of leaders who combine traditional skills in international relations and public health with a multidimensional perspective, political lens and renewed sense of collective accountability to tackle this metacrisis. Politically astute leaders can leverage diplomacy to build coalitions, share knowledge and negotiate agreements that advance global health objectives.^8^ Modern health diplomats blend knowledge from a wide range of disciplines and fields beyond their traditional domains of public health or medicine—for example, politics, economics, history, law, business, ethics, the environmental sciences and many other fields—adapting to the multidisciplinary and interconnected nature of global health challenges.^9^ Their roles extend beyond forging alliances and sharing information to include negotiating agreements with a balanced view of national interests and global needs and benefits. This comprehensive, multidimensional approach is critical for successfully navigating complex global health issues and advancing global health objectives.

## Strategic future directions

Developing future health leaders at global and regional levels, therefore, requires cultivating skills in negotiation, political analysis, communication, public and private engagement and cross-cultural understanding. A nuanced approach, employing tools such as political economy analysis, is required to navigate this complex terrain.

The conference on ‘International Politics, Leadership and Diplomacy for Health’ also emphasised the need for programmes that blend health expertise with political acumen, preparing leaders to address future health challenges. Such learning opportunities could be offered by a range of institutions, from schools of public health or medicine to those focusing on public policy, business, law, governance or the social sciences, from tailor-made training programmes to informal sessions at conferences. Therefore, to advance global public health in this complex scenario, we propose the following strategies:

1. *Articulate health as a political choice:* positioning health as a strategic and political opportunity is vital. Global and regional public and private sector leaders must recognise that investing in health is not merely a moral imperative but a pragmatic, strategic decision with far-reaching benefits; this requires speaking a common language that can resonate across sectors.^10^
2. *Move beyond traditional public health models:* merging public health evidence with insights into other disciplines can provide the right tools and competencies for health diplomacy. This multidisciplinary approach, encompassing the language and priorities of politicians and the public, is key to navigating the complex political landscape and aligning health initiatives with broader political and social objectives.
3. *Leverage emerging global dynamics:* the increased engagement and empowerment of low-income and middle-income countries and under-represented populations, in addition to the emergence of private sector and regional blocs like the African Union and Association of Southeast Asian Nations and private sector in global health discussions, present novel prospects for innovative forms of enhancing cohesion and strengthening leadership. Engaging with these emerging powers can create more balanced and effective global health governance structures when combating global health challenges.^11^
4. *Cultivate a new generation of global public health leaders*: there is an urgent need to provide learning opportunities for global public health professionals who can adeptly navigate the current political realities.^12^ These leaders should emerge from diverse regions and sectors, including civil society, government, academia and the private sector, to foster a multifaceted approach to global health challenges.

## Conclusion

As we navigate an era marked by complex health challenges and opportunities to address and redress them, the role of innovative learning opportunities becomes increasingly vital. Experiential learning approaches which incorporate role-playing and real-world scenarios, such as global health diplomacy training programmes and Model WHO Simulations, equip emerging leaders with the practical skills and insights needed for effective health diplomacy in practice.^13^ By embracing these dynamic educational techniques, we can better prepare health diplomats to handle the multifaceted demands of global health and contribute significantly to addressing the world’s pressing health issues. Additionally, providing opportunities for emerging leaders to engage in peer-to-peer mentorship and intergenerational learning with seasoned practitioners is crucial. Such experiences not only enrich their understanding but also foster a collaborative spirit essential for tackling global health challenges.

The current global health landscape also offers unique opportunities for reinvigorating global health diplomacy. Political will and leadership, instrumental in past public health successes, remain pivotal. The strategies outlined above provide a roadmap for leveraging political dynamics to advance global health objectives. It is crucial to invest in a new generation of leaders adept at navigating these complexities to achieve future progress.^12 14^ Despite the daunting nature of the current metacrisis, the potential for collective action driven by strategic political leadership has the potential to effect substantial improvements in global health and safeguard health futures.^4^ This is a pivotal moment for global health, demanding a concerted, innovative approach to ensure health remains a priority on the global stage.

## Data Availability

There are no data in this work.
